# We still fail to account for Mendel's observations

**DOI:** 10.1186/1742-4682-1-4

**Published:** 2004-08-16

**Authors:** John W Porteous

**Affiliations:** 1 Department of Molecular and Cell Biology, Institute of Medical Sciences, University of Aberdeen, Foresterhill, Aberdeen AB25 2ZD, Scotland, UK

## Abstract

**Background:**

The present article corrects common textbook accounts of Mendel's experiments by re-establishing what he wrote and how he accounted for his observations. It notes the long-established tests for the validity of any explanations that purport to explain observations obtained by experiment. Application of these tests to Mendel's paper shows that the arguments he used to explain his observations were internally consistent but were, on one crucial issue, implausible. The same tests are applied to the currently accepted explanation for Mendel's observations.

**Conclusions:**

The currently favoured explanation for Mendel's observations is untenable. It misrepresents Mendel, fails to distinguish between the parameters and the variables of any system of interacting components, its arguments are inconsistent, it repeats the implausibility in Mendel's paper, fails to give a rational explanation for his observed 3:1 trait ratio and cannot explain why this ratio is not always observed in experimental practice. A rational explanation for Mendel's observations is initiated. Readers are challenged to complete the process before a further article appears.

## 1. Background

We all talk, more or less knowingly, about Mendelian genetics. But four questions need to be asked and answered.

1. Do we understand Mendel's work?

To judge from nearly all modern accounts of genetics, we do not. Mendel's paper of 1866 has been persistently misrepresented ever since it was rescued from obscurity in 1900.

2. Do we teach our students a rational description of the inheritance of traits?

The answer is again no. Why? Because our current depiction of the inheritance of traits or characteristics is based on false statements, inconsistent arguments and an implausible assertion.

3. Does the current description of Mendelian genetics account for his observations of dominant and recessive traits?

No, for the reasons given in answering question 2.

4. Do we account rationally for Mendel's observation of a 3(dominant):1(recessive) trait ratio in some but not all of his experiments?

The answer is again no. The reasons will become clear in this article and its successor.

A survey of the relevant literature for the period from 1900 to 2003 shows that the various misrepresentations of Mendel's first paper [1] are of long standing. This is not the place to review all the accumulated historical evidence. The present article concentrates on demonstrating that the currently favoured depiction of elementary Mendelian genetics is untenable; it fails to achieve its intended purpose. A change in the concepts and notation for the interpretation (and teaching) of elementary genetics is suggested.

There are two long-established tests of the validity of any hypothesis or proposed explanation for the results observed by experiment. The first test asks: Are all the arguments employed consistent, one with all the others? The second test asks: Are all the proposed mechanisms plausible? Could they be confirmed by experiment, i.e. by a "real"experiment or by a logical "thought experiment". Both tests must be passed if the proposed explanations for the observations are to be accepted.

If judgement is being passed on work carried out in the distant past, allowance must be made for the availability or lack of availability of tests of plausibility at that time. On the other hand, we should not hesitate to criticise a current explanation that fails tests of plausibility that are now available but were not available in the past.

These two tests of validity (consistency and plausibility) will be applied to Mendel's explanation for his observations and to the currently favoured explanation for his observations.

We must first re-establish what experiments Mendel performed and what he wrote in his published accounts of these experiments in order to correct the various false textbook descriptions of Mendel's work. For this purpose it is necessary to study authentic reprints of his two papers [1,2]. The first paper is the one we are concerned with here; it was reprinted [3] and in a version [4] correcting several type-setting errors that occurred when Mendel's manuscript was set in typescript. The translation into English by Sherwood [5] avoided several errors in earlier attempts to translate Mendel's *Versuche *paper [1]. There may be other sound translations, but Sherwood's version is strongly recommended. It is accurate and also captures Mendel's literary style.

## 2. Mendel's experiments and his conclusions

### 2.1. Why did Mendel carry out his experiments?

Many earlier biologists had noted the appearance of hybrid plants but their findings did not show how hybrids arose, whether there was any regularity in their occurrence, or how their properties were related to those of their parents. Mendel showed that there was a general rule for the appearance of hybrid plants and that an exact relationship existed between the traits displayed by hybrids and those displayed by their parents. Hence the title of his first paper: *Versuche über Pflanzen-hybriden *(Investigations on plant hybrids).

### 2.2. Mendel's preliminary work and his conditions for successful experimentation

Mendel recognised five preconditions for success in his experiments on the origin of hybrids:

(i) He needed suitable plants for his experiments. He chose *Pisum sativum *(the edible pea plant) for most of his work because many established varieties were readily available; and because the flowers enclose the reproductive organs, so minimising accidental cross-fertilisation by insect-or air-borne pollen.

(ii) *Pisum sativum*, like all leguminosae, is androgynous. The flowers contain both male (pollen or sperm) and female (germinal or ova) cells and are therefore normally self-fertilising. This provided experimental advantages, as we shall see.

(iii) It was necessary to have stocks of true breeding plants for his cross-fertilisation experiments. He therefore spent much time establishing that 22 varieties of edible pea plants were in fact true breeding. He discarded those plants that were not true breeding before starting his experiments on hybridisation.

(iv) He had to ensure that any cross-fertilisations were strictly under his control. To achieve this control, he removed all the immature pollen-bearing stamens from a true-breeding pea plant that displayed a particular trait, e.g. green seeds, then transferred pollen to these emasculated flowers from another true breeding pea plant that displayed an alternative form of the same trait (e.g. yellow seeds).

(v) Success depended on meticulous enumeration of the occurrence of hybrids, and of alternative traits, in the populations of plants that arose from his cross-and self-fertilisation experiments; and on repetition of each cross-fertilisation and self-fertilisation experiment in order to obtain reliable, average, results. Table 1 reveals the magnitude of Mendel's undertaking and records his observations on the occurrence of hybrids, and of plants displaying either dominant or recessive traits (see further descriptions in the following section). Reciprocal crosses gave the same results; Mendel thus established that male and female sex cells contributed equally to the final outcomes.

**Table 1:** Mendel's novel observations summarised. Mendel demonstrated that crossing parental plants bearing alternative forms (*A*) and (*a*) of any one of seven traits generated a F1 population of plants (not shown) all of which were hybrids (*Aa*). Each of these F1 hybrid plants displayed only one of the two alternative parental traits, defined as the dominating trait (*A*). When these F1 hybrid plants were allowed to self-fertilise, the ratio of dominant to recessive traits in the F2 population was always close to 3:1.

Pairs of parental plants		Their F2 progeny	
Dominant traits (***A***)	Recessive traits (***a***)	Number of F2 plants examined	Dominant:recessive trait ratio in the F2 population

Green pods	Yellow pods	580	2.82:1
Axial flowers	Terminal flowers	858	3.14:1
Red flowers	White flowers	929	3.15:1
Long stems	Short stems	1064	2.84:1
Inflated pods	Constricted pods	1181	2.95:1
Round seeds	Wrinkled seeds	7324	2.96:1
Yellow seeds	Green seeds	8023	3.01:1

### 2.3. Bateson's notation for successive stages in breeding experiments

The following account uses the notation proposed by Bateson [6] for successive generations arising from sexual reproduction:-

P = the original male and female parental generations;

F1 = the first filial progeny population arising from crosses between plants of the P generation;

F2 = the second filial generation that arises from sexual reproduction by members of the F1 generation – and so on.

The advantage of Bateson's notation is that it does not depend on any preconceived ideas about the mechanisms of inheritance of traits during sexual reproduction. It can therefore be used to describe the stages in Mendel's experiments without misrepresenting any of his observations, arguments or conclusions.

### 2.4. Mendel's initial observations summarised

Table 1 shows the results of seven different cross-fertilisations between parental (P) plants displaying alternative forms of the same trait, e.g. red rather than white forms of the trait "flower colour"; all individual plants in the F1 population displayed only one of the two parental trait forms. Also shown are the results observed by Mendel when he allowed these F1 plants to self-fertilise; the ratio of (*A*) form to (*a*) form plants was, in every case, close to 3:1. Mendel also carried out experiments in which he cross-fertilised plants displaying concurrently two or three trait differences, and then recorded the occurrence of each trait in the F1 and F2 generations. These results are not shown here but they were consistent with the findings exemplified in Table 1. These initial findings led Mendel to a remarkable generalisation and a definition.

(i) All plants in the F1 population displayed only one of any two differing trait forms (*A*) and (*a*) displayed by the parental (P) plants.

(ii) He defined the trait form that was displayed in the F1 plants as *das dominirende Merkmal *(*A*) – the dominating trait *(A)*. He defined the alternative trait form, which did not appear in any of the F1 plants, as *das recessivem Merkmal (a) *– the recessive trait (*a*).

### 2.5. Further experiments

Mendel now faced the problem of explaining how the 3(dominant):1(recessive) trait ratio arose in the F2 population of plants (Table 1). In further experiments on each of the seven crosses shown in Table 1, he was able to show that those F2 plants he had identified by the symbol (*a*) were 'constant form' (true-breeding) plants; i.e., when they were allowed to self-fertilise, all their F3 progeny displayed the same parental trait (*a*).

On the other hand, when F2 plants initially identified by the symbol (*A*) were allowed to self-fertilise *some *proved to be 'constant form' plants because, when they were allowed to self-fertilise, they produced F3 progeny that again displayed this same parental trait (*A*). But other plants initially identified by the symbol (*A*) in the F2 population were not 'constant form' plants. Some of their F3 progeny did display the original parental trait (*A*). Other plants in the same F3 population displayed the alternative parental trait (*a*). Yet other plants in this F3 population were again not 'constant form' plants. They were like the F1 plants (their "grandparents") and like the F2 parents from which they were immediately derived. When they were allowed to self-fertilise, some of their progeny displayed the (*A*) form, some the (*a*) form of trait and some were again like the F1 plants. The experimental procedures Mendel used to make these distinctions are readily understood by reading a reprint of the original paper or a reliable translation.

Given this ability to distinguish, by experiment, between those plants initially designated (*A*) and those now designated (*Aa*), Mendel was able to state the average distribution of trait forms among the plants of the F2 population as (one dominant: two hybrid: one recessive) or, in his notation, *(A + *2*Aa + a)*; i.e. the 3:1 trait ratio factored into the proportions 1:2:1.

Mendel was now able to add a further generalisation: When F1 plants were allowed to self-fertilise, 1/4 of the F2 population displayed the 'constant form' parental trait *(A) *that was displayed by the F1 plants, 1/4 displayed the 'constant form' parental trait *(a) *that did not appear in any of the F1 plants (Table 1), and 1/2 were hybrids (*Aa*) that displayed only the dominant trait (*A*) but were not 'constant form' plants.

### 2.6. Mendel's notation

Mendel used upper case and lower case italicised letters throughout his paper to denote, by definition, dominant and recessive *traits*. Examples have already been given of the use of letters (*A*) and (*a*) when only one trait difference between parental plants was tested (Table 1). Mendel made similar use of the letters (*B*) and (*b*), (*C*) and (*c*) when he described experiments in which two or three trait differences were displayed concurrently.

For reasons given in Section 2.5 these single letters also designated what Mendel called 'constant forms' of traits. Plants displaying these traits were 'true-breeders'; they were the parental plants he used in cross-fertilisations (Table 1).

There is one further crucial feature of Mendel's single letter notation for 'constant form' traits. These letters (*A, a, B, b, C, c*) did not represent the structure or composition of the traits. All the traits shown in Table 1 obviously had complex compositions. But, irrespective of such complexity, each dominant trait was denoted by (*A*) and each recessive trait by (*a*) in Table 1. The traits were what Mendel could see with his own eyes. He distinguished a dominant trait from a recessive trait by qualitative observations. He was not concerned with and did not analyse the structural composition of the traits.

The letters (*A, a, B, b, C, c*) represented classes of traits – a dominant class represented by an upper case letter, and a recessive class of trait represented by the corresponding lower case letter (Table 1). It is necessary to recognise these facts if a rational explanation for Mendel's observation is to be obtained; and if gross misrepresentations of Mendel's paper are to be detected.

Why then did Mendel use a combination of letters (e.g. *Aa*) to represent hybrid plants? This will become clear in section 2.7.

### 2.7. Postulates and arguments; Mendel's explanations of his observations

Mendel accounted for the two generalisations (section 2.4) by the following postulates and arguments; they were based on his further experiments (section 2.5):-

**Figure 1:**
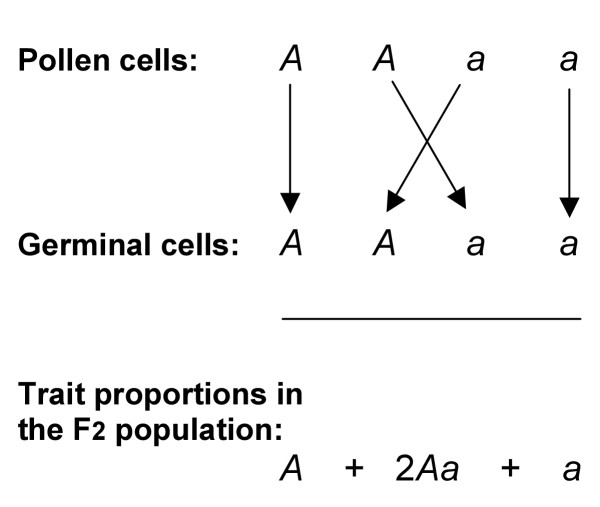
Mendel's diagrammatic explanation for the formation of the F2 population of plants produced by self-fertilisation of his F1 hybrids. Mendel proposed that F1 hybrids (*Aa*) contained a dominant trait (*A*) that was displayed and a recessive trait (*a*) that was not displayed. Self-fertilisation of F1 hybrids (*Aa*) then involved segregation of the component traits (*A*) and (*a*) into individual male pollen and female germinal cells, as shown in his diagram. Mendel proposed that if a male pollen cell carrying a trait (*A*) fertilised a female germinal cell carrying the same trait (*A*), the progeny would display trait (*A*). He used the analogous argument for the generation of progeny bearing trait (*a*). Only if male and female sex cells carried differing forms of a given trait (*A *or *a *but not both) would the progeny be hybrids (*Aa*). Thus random recombination of the segregated traits during self-fertilisation of hybrids would yield (on average) the F2 population of plants represented by the trait series (*A *+ 2*Aa *+ *a*) shown below Mendel's original diagram.

(1) All the F1 plants were hybrids (*Aa*) *in welcher beide Merkmale vereinigt sind *– in which both (parental) traits (*A *and *a*) were united; trait (*a*) was not displayed by these hybrids, so that these hybrids displayed what he had defined as the dominating trait (*A*) only.

(2) The traits (*A*) and (*a*) in the F1 hybrids (*Aa*) segregated into traits (*A*) and (*a*) during formation of the male pollen (sperm) cells and also during formation of the female germinal cells (ova). Thus, each pollen cell and each germinal cell carried only one trait – either (*A*) or (*a*) but not both.

(3) Fertilisation of one germinal cell by one pollen cell was a random event.

(4) When a pollen cell bearing trait (*A*) fertilised a germinal cell bearing the same trait (*A*), all their progeny displayed the trait (*A*). Likewise, when a pollen cell bearing a trait (*a*) fertilised a germinal cell bearing the same trait (*a*), all their progeny displayed the trait (*a*). But when a pollen cell bearing trait (*A*) fertilised a germinal cell bearing the alternative trait (*a*), the resulting plant was the hybrid (*Aa*); if the pollen cell displaying a trait (*a*) fertilised a germinal cell displaying the alternative trait (*A*), the outcome was again a hybrid (*Aa*). In either event, the hybrid (*Aa*) displayed only the dominant trait (*A*).

(5) Mendel illustrated these postulates and explanations in a diagram (Figure 1) showing the consequences of self-fertilisation of F1 hybrids (*Aa*), given that traits (*A*) and (*a*) in the hybrid (*Aa*) first segregated into individual pollen cells (sperm) and individual germinal cells (ova) before recombining, in random fashion, during formation of the F2 population. The arrows in Figure 1 represent the fertilising event.

Note two crucial points:-

(i) Mendel observed and recorded the occurrence of traits (*die Merkmale*) or the characters *(die Charaktere) *in his plants and their seeds, not the mechanisms underpinning these occurrences. These mechanisms could not have been investigated in 1866.

(ii) All Mendel's explanations were based solely on observations of the changes in the occurrence of alternative traits in successive populations that arose from cross-or self-fertilisations and back-crosses.

### 2.8. Comment

Mendel was a well-trained scientist [7], an astute thinker, a careful and systematic experimentalist, an expert hybridiser and an exemplary writer but he was not the first geneticist. That title should go, possibly, to Bateson [6,8,9] for advocating Mendel's experimental methods, for showing that Mendel's findings could be repeated in animals, and for emphasising that *combination*, *segregation *and *recombination *of *traits *during gametogenesis was the most important feature of Mendel's work. Moreover, Bateson realised [[6]; in a footnote on page 133] that the occurrence of alkaptonuria, one of the "Inborn Errors of Metabolism" first reported by Garrod [10-12], was an example of Mendelian recessivity of a trait or character. Bateson, incidentally, coined the word "genetics".

Another leading contender for the title "the first geneticist" was the Danish biologist, Johannsen [13,14], an equally enterprising experimentalist and astute thinker. Johannsen [14] was the first to define the term *"das gen*; (plural) *die gene" *as the determinant of a trait; he was also the first to make a clear distinction between the genotype *(der Anlagetypus) *and the phenotype *(der Erscheinungstypus*) on the basis of his experiments with self-fertilising bean plants. In Johannsen's experiments the weights of individual beans were the characteristics or traits. He had, in effect, repeated Mendel's experiments but by measuring a trait (individual bean weights in successive populations of plants) he was able to introduce three new concepts (gene, genotype and phenotype) that were the most significant, after Mendel's concepts of combination, segregation and recombination of traits during gametogenesis, in understanding the origin of genetic phenomena (the origin of changing traits).

Failures to recognise the significance of Johannsen's work [13,14] prevented the development of rational concepts in genetics for at least the first two decades of the 20^th ^century. This failure is, surprisingly, still evident in current depictions of elementary Mendelian genetics (Section 3).

### 2.9. The tests of validity applied to Mendel's explanation for his observations

It is clear that Mendel's experimental procedures (sections 2.2, 2.5) were sound; his notation was simple, unambiguous and consistently applied (section 2.6). His arguments (section 2.7) for a combination of traits in forming the F1 hybrids (*Aa*) are consistent with his arguments for the segregation of the component traits of the hybrid into separate gametes, and their random recombination in generating the F2 population (*A *+ 2*Aa *+ *a*). Mendel's arguments pass the test of consistency.

It is equally clear (but hitherto not noticed) that Mendel's explanations failed the test of plausibility. Mendel postulated that a F1 hybrid (*Aa*) was formed by combining the two differing traits (*A*) and (*a*) of their parents. He did not explain how a F1 hybrid (*Aa*) displayed only trait (*A*) and how it did not display trait (*a*), even when some F2 plants, like one of the two original parental (P) plants, did display trait (*a*). What explanation could we now give for this selective display of only one of two traits that are said to be combined in a hybrid?

It may be (and has been) argued by some that trait (*A*) was displayed by the hybrids (*Aa*) because (*A*) was a dominant trait and (*a*) was a recessive trait. Such statements do not even qualify as a circular argument. They are illogical. Such statements fail to distinguish between an arbitrary definition and a plausible explanation. Mendel's definition of a dominant trait should be seen as an arbitrary device that accounts for his observation (by experiment) that his hybrids (*Aa*) in the F2 populations (*A *+ 2*Aa *+ *a*) displayed trait (*A*) but not trait (*a*).

A word of caution is necessary. Mendel's formulation (*Aa*) for a hybrid was crucial in establishing his consistent arguments; it was also the basis for Bateson's recognition that the essential features of Mendel's work were the concepts of combination, segregation and recombination of alternative traits (i.e., components of the phenotype). If we now wish to replace Mendel's implausible formulation (*Aa*) for a hybrid by a plausible formulation, we face the prospect of abandoning the rest of Mendel's arguments. That is not to say that we abandon admiration for Mendel's work. For its time, it was unsurpassed and should be recognised as one of the important steps in the development of experimental procedures in what became known as genetics. We should take care not to misrepresent Mendel's experiments and his arguments. It will become clear that misrepresentations of Mendel's paper have served only to sustain untenable concepts in current biology.

In the post-Mendel era we assert that it is not components of the phenotype that segregate and recombine. It is the alleles (i.e., components of the genotype) that combine, segregate and recombine. May we then anticipate that modern explanations of Mendel's observations will pass the tests of consistency and plausibility?

## 3. Current accounts of elementary Mendelian genetics

### 3.1. Explanations of Mendel's observations

**Figure 2:**
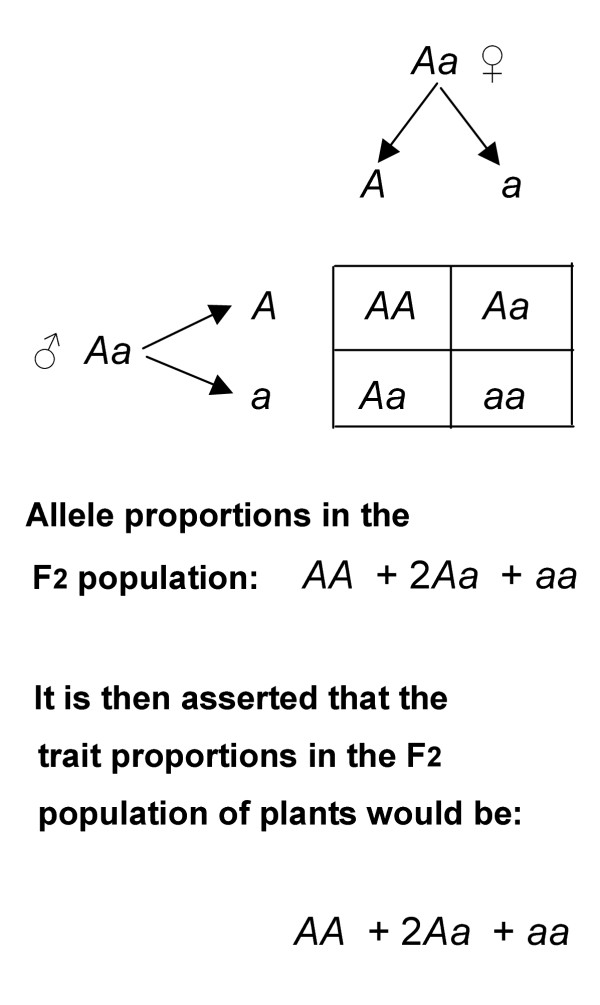
The currently favoured depiction of Mendelian inheritance following self-fertilisation of F1 hybrids represented by the allele pair (*Aa*). Section 3.1 of the text records the arguments commonly used in attempts to account for the alleged F2 trait series (*AA *+ 2*Aa *+ *aa*) and for Mendel's 3(dominant):1(recessive) trait ratio. Sections 3.2 and 3.3 discuss the faults in these arguments.

The currently favoured explanation for Mendelian heredity in general, and in particular for the occurrence of Mendel's 3(dominant):1(recessive) trait ratio, is shown in Figure 2.

The assertions and descriptions generally attached to Figure 2 are as follows.

(i) Mendel explained his experimental results by assuming that particles or factors (now called alleles) determined or specified the observed traits.

(ii) (*A*) is a dominant allele;*(a) *is a recessive allele.

(iii) The alleles in the male and female heterozygous somatic cells *(Aa) *segregate into separate gametes. Each gamete then contains only one dominant allele *(A) *or only one recessive allele *(a).*

(iv) Fertilisation is a completely random event. Given a large number of fertilisation events, the possible recombinations of alleles are those displayed in the four squares.

(v) Therefore the average distribution of the alleles at one diploid locus in the resulting progeny population of individual plants will be (*AA *+ 2*Aa *+ *aa*).

It is then argued that:

(vi) The dominant allele pair (*AA*) will give rise to a dominant trait (*AA*).

(vii) The recessive allele pair (*aa*) will give rise to a recessive trait (*aa*).

(viii) In the heterozygote (*Aa*), the recessive allele is ineffective, or is suppressed by the dominant allele (*A*), so that only the dominant allele (*A*) is expressed in the heterozygote. Expression from one (*A*) is as effective as that from two dominant alleles (*AA*). Thus the heterozygote (*Aa*) expresses a dominant trait.

(ix) Therefore the allele series (*AA *+ 2*Aa *+ *aa*) is expressed (in a population of the progeny plants, animals or cells) as the trait series (*AA + *2*Aa + aa*).

(x) This trait series gives rise to Mendel's 3(dominant):1(recessive) trait ratio (by the arguments in vi, vii, viii).

### 3.2. Faults in these currently favoured descriptions of Mendelian genetics

There are seven faults in the descriptions and arguments attached to Figure 2.

(i) Mendel is misrepresented; he did not assume that particles or factors specified the observed traits. It is historically inaccurate and scientifically misleading to suppose that he made any such assumption.

(ii) The letters (*A*) and (*a*) are Mendel's notation for dominant and recessive *traits *(Figure 1, Table 1). If we are to continue to discuss Mendelian genetics, these notations (and the nomenclature *dominant *and *recessive*) should refer to traits alone.

(iii) Figure 2 fails to distinguish between the components of the genotype and the components of the phenotype (Johannsen, Section 2.8) because it asserts that alleles are dominant or recessive; and uses the same notation (*A *and *a*) and the same nomenclature (*dominant *and *recessive*) for both.

(iv) Because we must not confuse alleles with traits, we could reasonably write an allele series as (*UU *+ 2*Uu *+ *uu*); this states that a given locus, in three genetically related diploid cells, comprises a pair of two normal alleles (*UU*), or one normal and one mutant allele (*Uu*), or a pair of two mutant alleles (*uu*). Mutations change the allele constitution or composition at a locus. The modern (non-Mendelian) notation (*AA + *2*Aa + aa*) in Section 3.1 (items vi, vii) then states explicitly that a dominant trait (*AA*) comprises two aliquots (*A *+ *A*) of some material substance or of two doses of dominance (*A + A*); likewise that a recessive trait (*aa*) is composed of two entitities (*a *+ *a*) or two doses of recessivity. This is simply not true. It was not true in Mendel's time and it is not true today. Furthermore, it is not what Mendel's notation meant. It was pointed out (Sections 2.5, 2.6) that Mendel's notation (*A*) and (*a*) distinguished *classes *of traits, specifically 'constant form' classes of traits (Table 1). To substitute (*AA*) for (*A*) and (*aa*) for (*a*) in a trait series is illogical and indicates a regrettable failure to read Mendel's paper with the care that should be given to one of the classic papers in biology.

(v) If the arguments attached to the homozygotes in Figure 2 are sound, they should also apply to the heterozygote. It is argued in Figure 2 that two dominant alleles (*AA*) generate a dominant trait (*AA*); and that two recessive alleles (*aa*) generate a recessive trait (*aa*). In other words, it is asserted that there is a direct, positive, linearly proportional (or additive) relationship between the allele constitution at a gene locus and the constitution of the trait expressed from that locus. If we are to be consistent, the same arguments should apply to the heterozygote (*Aa*).

On the contrary, the arguments in section 3.1 (item viii) state that one dominant allele (*A*) in a heterozygote (*Aa*) is as effective as two dominant alleles (*AA*) in the homozygote. The arguments in item (viii) are therefore inconsistent with arguments in items (vi) and (vii). Item (viii) also transfers Mendel's implausible assertion that a hybrid (*Aa*) displays only trait (*A*) to the equally implausible assertion that one allele (*A*) in a heterozygote (*Aa*) is as good as two such alleles in a homozygote (*AA*). The argument in item (viii) that allele (*a*) is ineffective is an extreme case; it is therefore not generally applicable. The alternative argument, that allele (*a*) in a heterozygote is suppressed by the dominant allele (*A*), lacks any experimental support or rational theoretical justification. Items vi, vii and viii attached to Figure 2 are arbitrary, irrational and implausible devices applied to the heterozygote alone; they seem to have been introduced solely in order to arrive at the desired result.

(vi) Figure 2 and the attached arguments thus fail to give rational explanations for the occurrence of dominant and recessive traits and for Mendel's 3(dominant):1(recessive) trait ratio.

(vii) Figure 2 does not and cannot account for the observation that dominance and recessivity are not observed for all traits. The assertion in Figure 2 that the alleles are themselves "dominant" or "recessive" (and thus determine that traits are dominant or recessive) conflicts with inability of Figure 2 to explain why dominant and recessive traits are not always observed; nor does Figure 2 account for the observation that, when dominance and recessivity do occur, they do not always exhibit a 3:1 trait ratio.

### 3.3. Comments on these faults

It is necessary to restate fault (iii) in section 3.2 in more widely applicable terms. It is illegitimate to use the same notation and nomenclature for a *parameter *and a *variable *in the same system. Parameters are those components of any system that are directly accessible to the experimentalist; they can be changed and *maintained *by the experimentalist at the new value, at least for the duration of an experiment. Variables are those components of the same system that are not directly accessible to the experimentalist; they can be changed and maintained at a new value only by making a finite change in at least one parameter of the system or of its immediate environment. The magnitudes of individual variables, in any system, respond to changes in the magnitude of one or more parameters of the system or of the immediate environment.

In the case under discussion, the alleles are parameters (and part of the genotype); the traits are variables (and part of the phenotype). If the parameters and variables of any system of interacting components are represented by the same notation and the same nomenclature, confusion will inevitably result – as illustrated by Figure 2, by the assertions (i) and (ii) and by the false arguments (vi) to (x). Traits may be dominant or recessive [1]; alleles cannot also be dominant or recessive.

Figure 2, and the arguments attached to it, fail all tests of consistency and plausibility (Section 2.9); they also fail the test of historical accuracy.

### 3.4. Another example of the improper transfer of dominance/recessivity from traits to alleles

The primary error in Figure 2 is the illegitimate transfer of Mendel's terms "dominant" and "recessive" from traits (variables) to alleles (parameters), followed immediately by the reverse (and perverse) argument that the traits specified by the alleles must be dominant or recessive *because the alleles are dominant or recessive*. This habit is unscientific. It also occurs in discussion of mutations of non-catalytic proteins.

When haemoglobin A (HbA) is mutated to the sickle cell haemoglobin (HbS), the three possible trait forms are correctly depicted as follows:

(A/A) – the homologous, *normal/normal *protein, condition;

(A/S) – the heterologous, *normal/mutant *protein (sickle cell), condition;

(S/S) – the homologous, *mutant/mutant *protein, condition.

Contrast these depictions with those sometimes found:

(A/A) – the *dominant *condition;

(A/S) – the *sickle cell *condition;

(S/S) – the *recessive *condition.

These latter statements depend solely on the illegitimate transfer of Mendel's terms *dominant *and *recessive *from traits (variables) to alleles (parameters) and the contention that, if alleles are themselves dominant or recessive, their expressed traits must always be dominant or recessive. If changes in the composition of non-catalytic proteins do explain the occurrence of Mendel's dominant and recessive traits, we require a demonstration that does not depend on these illogical notions.

The sickle cell trait (A/S) in humans is significantly different from the normal trait (A/A).

Those carrying the sickle cell (A/S) condition enjoy an advantage in areas *where malaria is endemic*. They do not die *from malaria *as frequently as those in the population with the (A/A) condition. The sickle cell condition (A/S) is debilitating but, provided it is not too debilitating, the frequency in the local population of those carrying the (A/S) protein pair is greater than it would be in malaria-free areas.

This higher frequency of the sickle-cell (A/S) condition in areas where malaria is endemic is often said to be an example of "over-dominance". The term "over-dominance" is inappropriate. It presumably arose from the illegitimate transfer of the terms *dominant *and *recessive *noted above. The appropriate term is "heterozygous superiority". The "superiority" indicates the better chance of surviving in regions where malaria is endemic.

## 4. Conclusions: beginning a rational explanation for Mendel's observations

The illegitimate use in Figure 2 of the same notation (*A *and *a*), and the same nomenclature (dominant and recessive), to describe an allele series and a trait series can be traced to Sutton [15]. Sutton asserted that the proportions of the chromosome pairs in the F2 population "would be expressed by the formula *AA*:2*Aa*:*aa *which is the same as that given for any character in the Mendelian case." Mendel's expression (*A *+ 2*Aa *+ *a*) gave the proportions of characters in his F2 population as *A*:2*Aa*:*a*. Sutton gave no justification for rewriting these proportions in the form *AA*:2*Aa*:*aa*. By writing the expression for chromosome pairs as *AA*:2*Aa*:*aa *and the expression for the proportions of F2 characters as *AA*:2*Aa*:*aa*, Sutton established a direct, one-for-one, relationship between pairs of chromosomes and the traits arising from them. This false relationship also persists in the currently favoured depiction of Mendelian genetics (Figure 2). Sutton's notation for pairs of chromosomes (*AA*:2*Aa*:*aa*) was later transferred to pairs of alleles (what Sutton described as subunits of the chromosomes).

It would be easy to blame Sutton for our present confusions. We should remember that Sutton, and those in the early years of the 20^th ^Century who copied his error, were struggling to understand the hereditary origin of traits.

We may more reasonably ask: Why, one hundred years later, are these obvious errors still one of the features of Figure 2? Have these errors not been noticed before or, if they have been noticed, why they have not been corrected? Why also has the inconsistency and the implausibility of the arguments attached to Figure 2 not been noticed or corrected? Why (in both of the examples given in sections 3.3 and 3.4) are alleles (components of the genotype) not distinguished, as they surely should be in genetics, from traits (components of the phenotype) by using different notations and nomenclatures for each?

Traits (variables) may be dominant or recessive, as defined by Mendel. Alleles (parameters) are, always have been, and can only be normal or abnormal (mutant). Harris (pages 143–157 in reference [16]), for example, referred consistently to normal and abnormal alleles (not to dominant and recessive alleles), whereas, as noted above, alkaptonuria was a Mendelian recessive trait or character (page 133 in reference [6]; page 19 in reference [16]).

A review of 13 textbooks of genetics showed that in 12 instances, dominance and recessivity were defined specifically as properties of genes or alleles. These texts, published between 1982 and 2002, were intended for student use; their definitions of dominance and recessivity ignore Mendel's definition of dominance and recessivity as properties of the traits (sections 2.4, 2.5, 2.6, 2.7); they take no account of the need to distinguish between the parameters and variables of a system of interacting components (section 3.2). In one of these 12 texts, it was further claimed that: "Mendel proposed the existence of what he called particulate unit factors for each trait". In another, that: "Mendel realised that some genes (dominant genes) expressed themselves when present in only one copy". In a third that: "Mendel imagined that during the formation of pollen and egg cells, the two copies of each gene in parents segregate". Of these three quoted texts: The first misrepresented Mendel; he did not "propose the existence of particulate unit factors for each trait". The second misrepresented Mendel by transferring his term *dominirende *("dominating") from traits to genes; the second and the third quoted texts ignored the fact that the term "*das gen*" (plural "*die gene*") was first used and its role as the determinant of traits postulated by Johannsen, 43 years after Mendel's paper was published (Section 2.8); Mendel did not mention the word gene (Section 3.2). Of the 13 texts examined, only one gave a definition of dominance and recessivity that would have been recognised by Mendel. Even so, this author contradicted his correct definition of dominance and recessivity as properties of components of the *phenotype *by giving an explanation of elementary Mendelian genetics that employed Figure 2 and its associated arguments. All 13 of the texts examined ignored or contradicted the verifiable historical evidence (sections 2.2–2.7) and failed to make the obligatory distinction between the functions of alleles and the properties of traits.

The correct nomenclature for alleles used by Harris (pages 143–157 in reference [16]) is, unfortunately, rarely if ever employed by other authors. Pasternak [17], for example, accepted that "in strict genetic terms, dominance and recessivity are descriptions of the phenotype and not of the genes." but then continued: "However, few textbooks bother to make the distinction, because it was both convenient and highly ingrained for geneticists and others to refer to dominant and recessive alleles." Ingrained it may be, convenient (and scientifically legitimate) it is not.

If we continue to propose Figure 2 and the attached arguments as an explanation of Mendel's work, we deceive ourselves and encourage irrational thinking in our students at a time in their education when they are most vulnerable. It is extraordinary that an "explanation", like Figure 2, should still be found in textbooks intended for student instruction; it exposes our own confusion but explains nothing of scientific value in genetics. Any student who criticised Figure 2 and the attached arguments in an answer to an examination question would have shown commendable scientific insight but, according to current teaching, would be deemed to have failed that question.

Barker [18], writing on another topic, suggested that it might take 50 rather than 25 years for textbooks "to get it right". On the evidence presented here, Barker was too optimistic. The four errors introduced by Sutton [15] remain uncorrected (Figure 2) 100 years later. To be fair to authors of textbooks of genetics, every author inevitably relies on what has been written by preceding authors. However that may be, we are faced with an uncomfortable question. Are we content to continue to deceive ourselves, to give our students a false picture of what Mendel achieved, and to provide them with untenable 'explanation' of his remarkable observations (Figure 2)? Presumably not, especially when we can very easily begin, in this article, to construct a rational explanation for Mendel's observations and for other observations of current interest in genetics.

A fresh approach to the origins of dominant and recessive traits is needed. As a first step, we need to represent normal and mutant *alleles *by symbols that differ from those (*A, a, B, b, C, c*) used by Mendel to represent *traits*. We must replace symbols (*A *and *a*) for *alleles *in Figure 2 by quite different symbols; e.g. (*U*) to represent a normal allele, *not *a "dominant allele"; and (*u*) to represent a mutant or abnormal allele, *not *a "recessive allele"*. *The F2 allele series in Figure 2 would then be, on average, *UU *+ 2*Uu *+ *uu.*

Similarly, the *trait *series in Figure 2 must be replaced by Mendel's notation *(A *+ 2*Aa *+ *a) *because, as explained earlier, Mendel was concerned (as we are, first and foremost) only with understanding the origin of two *classes *of trait – the dominant class (*A*) and recessive class (*a*). We will later be concerned with the quantitative composition of traits.

We have, however, already identified an implausibility in Mendel's notation (*Aa*) for a hybrid that, allegedly, displayed the trait (*A*). An implausibility, like an inconsistency, must be eliminated if we are to arrive at an internally consistent and plausible account of Mendel's observations. The implausible notation (*Aa*) can be eliminated by replacing it by the single symbol (*H*) for a hybrid.

We have now adopted a stance that, in sharp contrast to Figure 2, distinguishes clearly between determinants and that which is determined. We have allocated a nomenclature and notation for alleles that is distinct from that allocated to traits. We have differentiated clearly between the *parameters *of the system (in this particular case, the components of the genotype) and the *variables *of the system (in this particular case, the components of the phenotype).

Mendel found, by experiment, that the proportions of different plant forms in his F2 populations were 1(dominant trait):2(hybrids):1(recessive trait) or, in his notation, (*A *+ 2*Aa *+ *a*). Replacing Mendel's notation (*Aa*) for a hybrid by the single symbol (*H*) does not alter Mendel's experimental observation of the proportions of trait forms in the F2 populations (section 2.5). It does mean that we can avoid Mendel's implausible postulate that, although recessive trait plants did display trait (*a*), his hybrids (*Aa*) did not. We have, of course, to discover an experimentally verifiable mechanism that would explain why hybrids (*H*) display a trait that is sometimes indistinguishable and sometimes distinguishable from trait (*A*).

Our remaining task is to explain rationally how this series of *normal *and *mutant *alleles *(UU *+ 2*Uu *+ *uu) *in the F2 population is expressed as the trait classes (*A *+ 2*H *+ *a*) in that population, where all that we have done is to replace Mendel's implausible (*Aa*) by a plausible (*H*). Note also that we have now also eliminated the illegitimate use of paired symbols for Mendel's dominant (*A*) and recessive (*a*) traits.

Most of the clues that facilitate this task are present in this article. One clue is missing, but it can be inferred by asking how one allegedly dominant allele (*U*) in a heterozygote (*Uu*) could be as effective as two such alleles (*UU*) in a homozygote.

A further article will provide the answers, but in the interval readers may like to rise to the challenge of explaining: (1) how *dominant and recessive traits *arise from *normal and mutant alleles*, and (2) why Mendel's 3:1 trait ratio, though not uncommon, does not always occur.
